# MHC Class I-Presented T Cell Epitopes Identified by Immunoproteomics Analysis Are Targets for a Cross Reactive Influenza-Specific T Cell Response

**DOI:** 10.1371/journal.pone.0048484

**Published:** 2012-11-07

**Authors:** James S. Testa, Vivekananda Shetty, Julie Hafner, Zacharie Nickens, Shivali Kamal, Gomathinayagam Sinnathamby, Ramila Philip

**Affiliations:** Immunotope, Inc., Doylestown, Pennsylvania, United States of America; Tulane University, United States of America

## Abstract

Influenza virus infection and the resulting complications are a significant global public health problem. Improving humoral immunity to influenza is the target of current conventional influenza vaccines, however, these are generally not cross-protective. On the contrary, cell-mediated immunity generated by primary influenza infection provides substantial protection against serologically distinct viruses due to recognition of cross-reactive T cell epitopes, often from internal viral proteins conserved between viral subtypes. Efforts are underway to develop a universal flu vaccine that would stimulate both the humoral and cellular immune responses leading to long-lived memory. Such a universal vaccine should target conserved influenza virus antibody and T cell epitopes that do not vary from strain to strain. In the last decade, immunoproteomics, or the direct identification of HLA class I presented epitopes, has emerged as an alternative to the motif prediction method for the identification of T cell epitopes. In this study, we used this method to uncover several cross-specific MHC class I specific T cell epitopes naturally presented by influenza A-infected cells. These conserved T cell epitopes, when combined with a cross-reactive antibody epitope from the ectodomain of influenza M2, generate cross-strain specific cell mediated and humoral immunity. Overall, we have demonstrated that conserved epitope-specific CTLs could recognize multiple influenza strain infected target cells and, when combined with a universal antibody epitope, could generate virus specific humoral and T cell responses, a step toward a universal vaccine concept. These epitopes also have potential as new tools to characterize T cell immunity in influenza infection, and may serve as part of a universal vaccine candidate complementary to current vaccines.

## Introduction

Influenza virus is a significant public health problem internationally, causing three to five million cases of severe illness, and an estimated 250,000 to 500,000 deaths annually [1]. Influenza virus is a member of orthomixovirdae and its genome is comprised of eight segments of negative single stranded RNA [2]. Viral strains are divided into A, B, and C viruses and differ serologically only between the HA and NA proteins [3]. Influenza constantly modifies these glycoproteins by implementing antigenic drift and shift [4], which is the main reason for influenza pandemics and the requirement for seasonal vaccines. The immune response to influenza is governed by both innate and adaptive immunity and has been well-studied. The humoral arm of the adaptive immune response utilizes secretory IgA and IgM to provide protection against the establishment of initial infection, while IgG acts to neutralize newly replicating virus [5], [6]. Improving humoral immunity to influenza is the target of current conventional influenza vaccines, however, they are generally not cross-protective [7]. Cell-mediated immunity, on the other hand, as elicited by major histocompatibility complex (MHC) class I-restricted CD8+ cytotoxic T lymphocytes (CTLs), plays a central role in controlling influenza virus infection [8]–[11] [12]. Indeed, cell-mediated immunity generated by primary influenza infection provides substantial protection against serologically distinct viruses due to the recognition of cross-reactive epitopes, often from internal viral proteins conserved between viral subtypes [13]–[15].

Tremendous efforts are underway to develop a universal flu vaccine that would work against all types of influenza. Such a universal vaccine should target conserved influenza virus antibody and T cell epitopes that do not vary from strain to strain [16]. Unfortunately, most conserved viral proteins lie within the virus, out of reach of antibodies. With a focus on antibody-mediated protection, attempts are being made to use a part of the external M2 protein (M2e) in addition to the HA stalk region, both of which are highly conserved among human influenza type A viruses [17]–[20]. Primarily, motif prediction methodology is used to identify shared T cell epitopes [21], however, there is evidence in the literature that a high number of predicted epitopes are not processed and presented by infected cells [22]. In the last decade, immunoproteomics, or the direct identification of HLA class I presented epitopes from infected cells, has emerged as an alternative to the motif prediction method [23]–[26]. These analyses are generally based on the isolation of the HLA-peptide complexes, elution of bound peptides from HLA molecules, and examination using mass spectrometry [25], [27]–[31].

In this study, we have identified T cell epitopes naturally presented on influenza A-infected cells using immunoproteomics. By surveying the MHC/peptide complexes present on the surface of influenza A-infected cells, we have identified novel conserved epitopes from various surface and internal influenza proteins. These shared T cell epitopes, when combined with a cross-reactive antibody epitope, such as the M2e peptide, generate cross-strain specific cell mediated and humoral immunity, which would be a positive step towards a potential universal vaccine for influenza infection.

## Methods

### Ethics Statement

All of the animal experiments were conducted according to the recommendations in the Guide for the Care and Use of Laboratory Animals of the National Institutes of Health. The protocol was approved by the Lampire Biologicals Institutional Animal Care and Use Committee (IACUC Permit - Core Rodent Immunotope 2012 Approved 5-31-2012).

### Mice

Six to eight-week old female HLA-A2 transgenic mice were purchased from Taconic (Strain HLA-A2.1, CB6F1-Tg(HLA-A*0201/H2-Kb)A*0201).

### Virus

Non-irradiated, sucrose-purified influenza virus strains A/PR/8/34(H1N1) (PR8), and A X:31, A Aichi/68(H3N2) (X31) were purchased from Charles River laboratories. Influenza strain A2/Japan/305/57(H2N2) (JAP) was a generous gift from Laurence Eisenlohr and used as unpurified allantoic fluid.

### Cells

HepG2, hepatoma cells, JY, EBV transformed lymphoblastoid B cells, and T2, lymphoblasts, were obtained from ATCC. HepG2 were maintained in DMEM:F12 medium while JY, and T2 were maintained in RPMI 1640 (Mediatech). All culture medium was supplemented with 10% fetal bovine serum, L-glutamine (300 mg/mL), non-essential amino acids (1× concentration), 0.5 mM sodium pyruvate, penicillin and streptomycin (1× concentration, supplements were purchased from Mediatech) [complete medium]. All cell lines were maintained at 37°C in a humidified incubator with 5% CO2.

Dendritic cells (DC) were generated from leukophereses obtained from HLA-A2+ healthy donors (Research Blood Components, LLC) as previously described [32]. Briefly, peripheral blood mononuclear cells (PBMC) were purified according to standard methods and, after overnight culture, were treated with 100 ng/mL IL-4 and 25 ng/mL Granulocyte Monocyte Colony Stimulating Factor (GM-CSF) (both cytokines from Peprotech or eBiosciences) for six days prior to their infection with influenza virus.

### Analysis of Infection

To verify infection, HepG2, JY, and monocyte-derived human DC were pulsed with 1000HAU/1×10^6^ cells of purified virus (PR8, X31) or 200HAU/1×10^6^ cells of unpurified virus (JAP) in PBS+.1% BSA for approximately 1 hr at 37°C. 16 hrs post-infection, cells were stained for intracellular nucleoprotein (NP) and were analyzed using a Guava flow cytometer (Millipore). Cells were stained with the primary antibody, HB65 (a generous gift from Dr. Laurence Eisenlohr), followed by FITC labeled anti-mouse secondary antibody (Invitrogen). The stained cells were analyzed using the Guava flow cytometer accompanying software.

### Isolation, Purification and Fractionation of MHC Class I Bound Peptides

JY cells were grown to about 1×10^9^ cells. Monocyte-derived DCs were generated over a period of six days to about 5×10^8^ cells. These cells were then infected with PR8 at 1000HAU/1×10^6^ cells. Influenza-infected cells were then processed as previously described [33]. Briefly, cell lysates were prepared in buffer containing 1% NP40 (IBI-Scientific), and MHC/peptide complexes were isolated by immunoaffinity chromatography using W6/32 antibody- (monoclonal antibody recognizing pan MHC class I molecule) coated protein A/G beads (UltraLink Immobilized Protein A/G, Pierce). The bound MHC/peptide complexes were eluted from the beads by the addition of 10% acetic acid. The eluate was heated at 85°C for 15 min to dissociate the bound peptides from the MHC molecules. Peptides were separated from the antibody by centrifugation using Amicon Ultra-3 kDa molecular mass cutoff membrane filters (Millipore). The purified peptide mixture was fractionated using a C-18 reversed phase (RP) column on an offline ultimate 3000 HPLC (Dionex).

### Mass Spectrometry Analysis

Mass spectrometry experiments were carried out using an Orbitrap (Thermo) instrument interfaced with nano ultimate HPLC (Dionex). RP-HPLC purified peptide fractions were injected individually into the LC–MS/MS system to identify the sequences of the peptides. The peptides were analyzed using a Data-Dependent method and the acquired spectra data were searched against the influenza protein database using Proteome Discoverer (Thermo) to interpret data and derive peptide sequences. For validation, synthetic peptides were obtained from Genscript Corporation and subjected to LC-MS/MS analysis under identical experimental conditions. Their sequences were confirmed based on their MS/MS data with that of their synthetic analogs.

**Figure 1:**
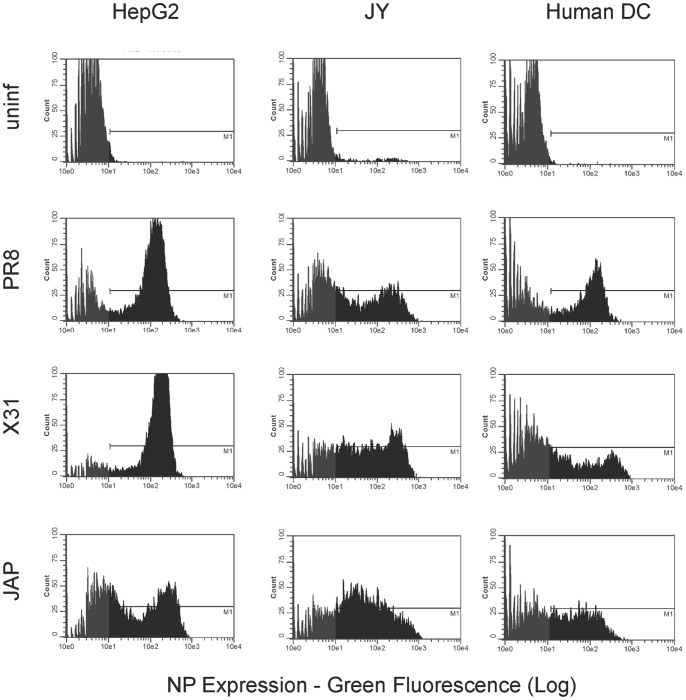
Influenza strain infection analysis. HepG2, JY, and monocyte-derived human DC were pulsed with 1000HAU/1×10^6^ cells of purified virus (PR8, X31) or 200HAU/1×10^6^ cells of unpurified virus (JAP) in PBS+.1% BSA for approximately 1 hr at 37°C. Cells were fixed and intracellularly stained for influenza NP after overnight incubation.

### Generation of CTL

Peptide-specific T cells were generated as previously described [32]. Briefly, Peripheral blood mononuclear cells (PBMC) were purified from heparinized blood obtained from healthy HLA-A2+ donors (Research Blood Components, LLC.) using lymphocyte separation medium (Mediatech). PBMC were pulsed with 50 µg/mL synthetic peptide and 1.5 µg/mL human β2-microglobulin (EMD Biosciences) overnight followed by stimulation with IL-7 at 5 ng/mL, Keyhole Limpet Hemocyanin (KLH, Sigma-Aldrich) at 5 µg/mL, GM-CSF at 25 ng/mL and IL-4 at 50 ng/mL (all cytokines and growth factors were purchased from Peprotech or eBiosciences). Two weeks after initial stimulation, T cells were restimulated with autologous CD4+/CD8+ T cell-depleted PBMCs pulsed with synthetic peptide at 10 µg/mL and 1.5 µg/mL human β2-microglobulin in complete medium containing 5 ng/mL IL-15, 12.5 ng/mL GM-CSF, and 10 U/mL IL-2 for 7 days. Restimulation was repeated three times at the indicated time intervals prior to use in ELISpot assays.

### ELISpot Assay

Antigen stimulated interferon-γ (IFN-γ) release as a measure of CTL activation was assayed using an ELISpot assay kit (BD-Pharmingen) according to the manufacturer’s instructions. Typically, a fixed number of various target cells and effector cells at an effector to target ratio of 100∶1 for PBMC or 10∶1 for murine splenocytes were cultured in replicate wells overnight. Spots were quantitated using an ELISpot Reader System (AID). Results are presented as the number of interferon-γ producing cells per 1×10^6^ PBMCs/splenocytes. Each assay was performed with PBMCs from at least three different healthy HLA-A2+ donors. Error bars represent SEM of experimental replicates.

**Figure 2:**
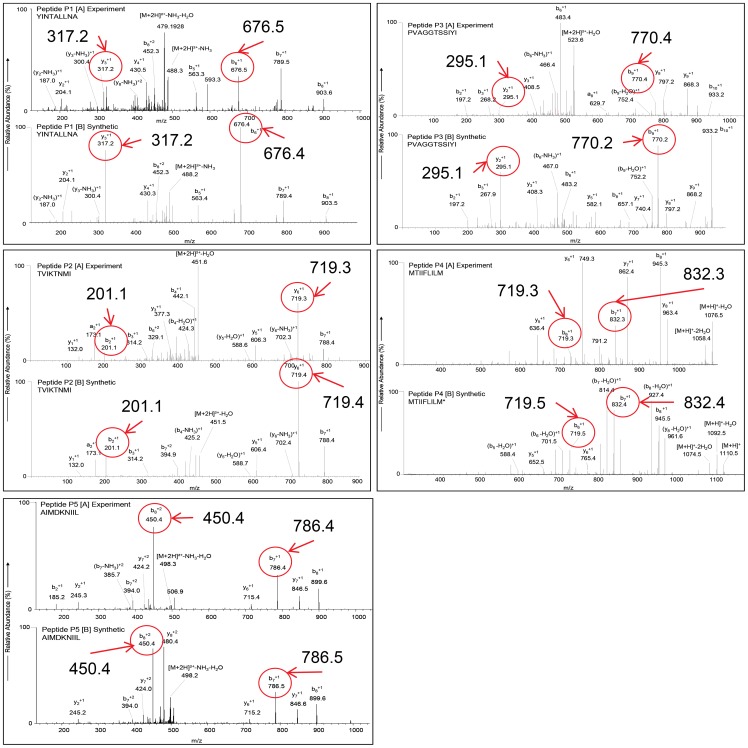
Synthetic Validation of Peptides. MS/MS spectra of experimentally identified MHC peptides [P1A–P5A] and their synthetic analogs [P1B–P5B]. Example fragment masses that match are denoted (circled).

**Table 1:** List of MHC class I presented influezna virus specific peptides identified by immunoproteomics analysis.

Peptide Number	Peptide	Protein	Influenza strain	Accession ID	Motif	m/z	Charge	Xcorr	Score	
									Bimas†	SYFPEITHI*
P1	YINTALLNA	polymerase PA	H5N1	gi168805480	A2	496.8	2	1.8	7.2	20
		polymerase PA	H1N1	A3DRP7						
		polymerase PA	H3N2	O91742						
		polymerase PA	H3N8	P13169						
		polymerase PA	H4N6	P13172						
P2	TVIKTNMI	polymerase PB1	H5N1	gi172053048	A2	460.3	2	2.1	–	–
		PB1 polymerase subunit	H9N2	Q9Q0S3						
		RNA-directed RNA polymerase catalytic subunit	H7N3	E9P6L9						
		RNA-directed RNA polymerase catalytic subunit	H1N1	A6M777						
P3	PVAGGTSSIYI	polymerase PB2	H3N2	gi215480628	A2	603.3	2	2.4	1.4	19
		polymerase PB2	H1N2	Q75TA1						
P4	MTIIFLILM	hemagglutinin	H2N3	gi257123295	A2	1094.6	1	1.7	1.0	15
P5	AIMDKNIIL	nonstructural protein 1	H1N1	gi138898	A2	515.8	2	2.1	18.3	23
		nonstructural protein 1	H3N8	Q77ZM3						
		nonstructural protein 1	H2N8	O57276						
		nonstructural protein 1	H5N9	O41649						
		nonstructural protein 1	H4N6	Q6LDH2						

† Calcluated score based on halftime for dissociation of the peptide from HLA molecule [51].

* Binding affinity scores calculated using SYFPEITHI database [52].

### *In vivo* Analysis

HLA-A2 transgenic mouse experiments were conducted at Lampire Biologicals. Their initial inoculation consisted of a mixture of pooled free peptide in PBS plus Montanide ISA 51 (Seppic) (50∶50 emulsion). In addition to the T cell epitopes, an antibody epitope derived from the ectodomain of influenza Matrix 2 protein (pM2e, MSLLTEVETPTRNEWESRSSDSSD) [17], [18] at 10 µg each was selectively injected. Mice were injected at two sites; i.d. near the base of the tail and s.c. on the flank. Injections were repeated two more times at 10-day intervals. One week after the third inoculation, mice were bled, sacrificed, and spleens were harvested for analysis.

Spleens were homogenized and RBCs lysed. Next, cells were restimulated *in vitro* with peptides as previously described [34] and used in an ELISpot assay at an E:T ratio of 10∶1 with uninfected or infected HepG2 or JY cells and peptide-loaded T2 cells as targets.

Flow cytometry was also carried out on *in vitro* stimulated splenocytes as previously described [35], [36]. After overnight incubation, cells were stained with directly conjugated antibodies against murine CD8 and CD107a (eBiosciences). Samples were analyzed using a Guava flow cytometer and accompanying software by gating CD8+ cells and graphing the mean fluorescence of CD107a for each group.

**Figure 3:**
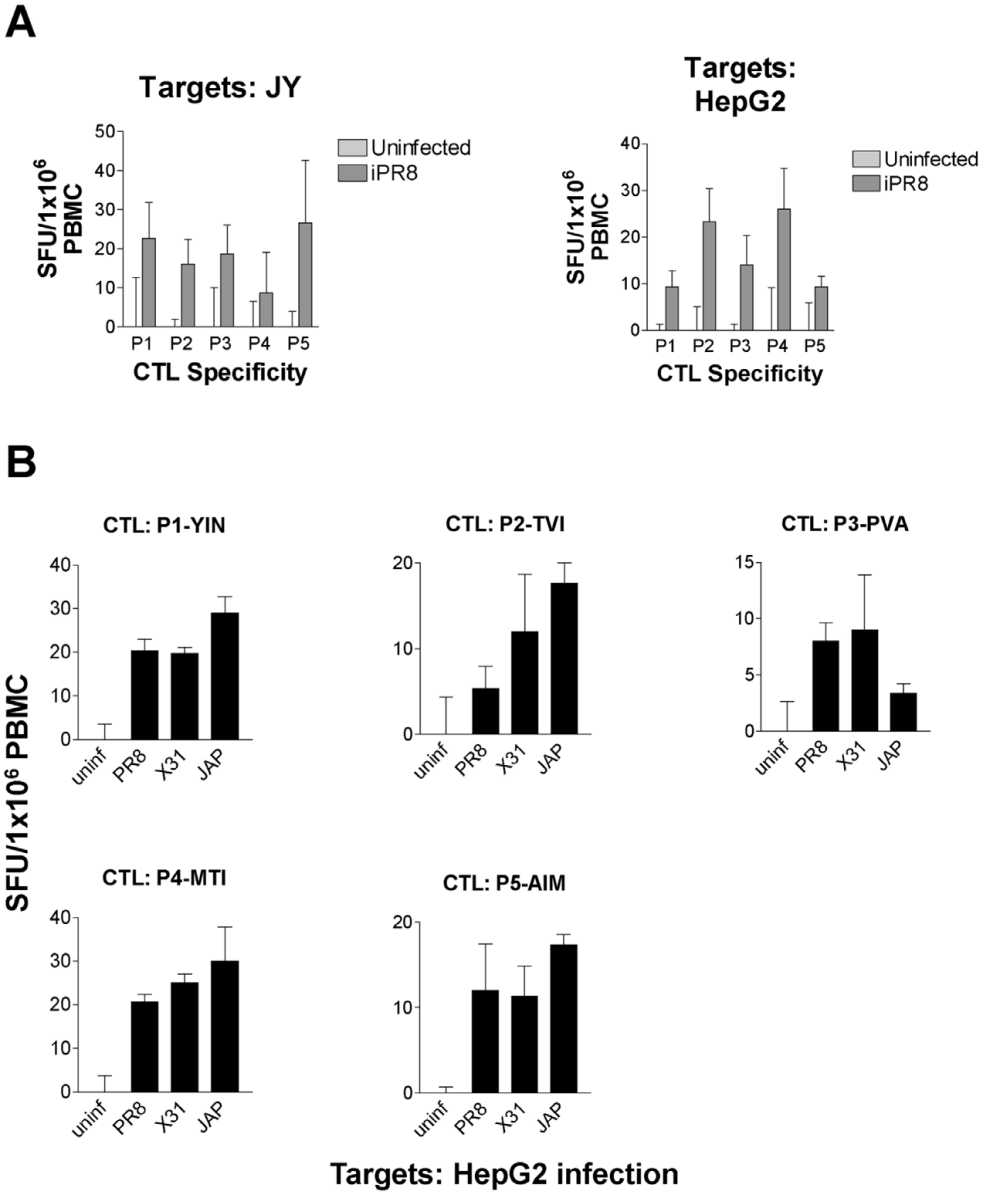
CTLs generated *in vitro* with influenza epitopes are specific and cross-reactive. (A) HepG2 and JY cells were left untreated or infected with PR8 and used as targets in an ELISpot assay with CTLs that were generated from HLA-A2+ PBMCs against specific peptides. (B) HepG2 cells were infected with PR8, X-31, or JAP and used as targets in an ELISpot assay. Results were normalized against uninfected controls.

### Anti-M2e Analyses

Serum samples from terminal bleeds were analyzed for IgG specific for M2e peptide using standard ELISA techniques. Additionally, serum samples were used to measure the presence of the M2e antibody epitope on the surface of infected cells. HepG2 cells were infected with PR8, X31, and JAP viruses as described above. After overnight incubation, cells were stained with serum samples at a 1∶50 dilution followed by FITC-labeled anti-mouse IgG (Invitrogen) secondary antibody. Samples were analyzed using a Guava flow cytometer and accompanying software.

**Figure 4:**
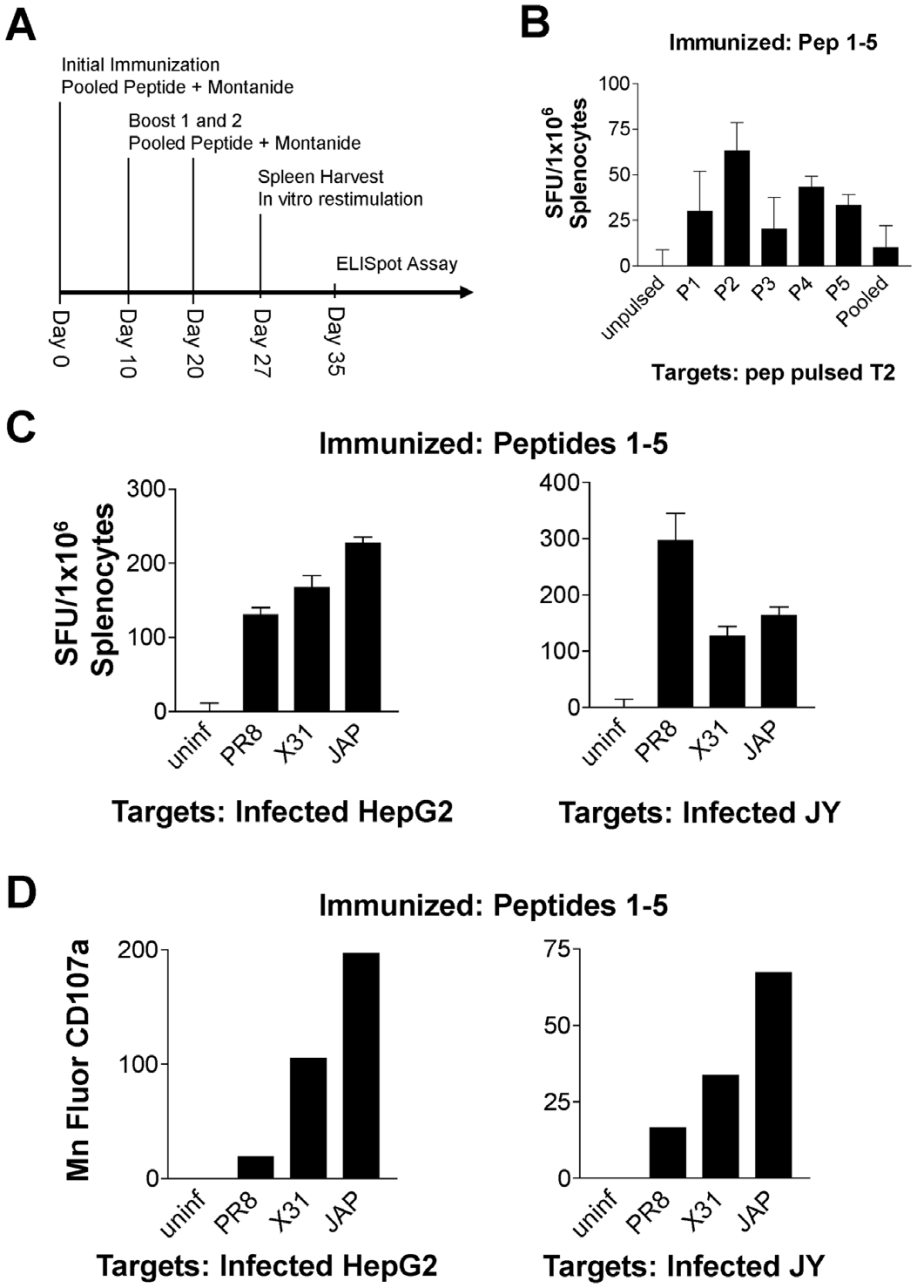
CTLs generated with influenza epitopes *in vivo* using humanized mice are specific and cross-reactive. (A) Immunization scheme for peptide injections. (B) T2 cells were pulsed with peptide and used as targets in an ELISpot assay with CTLs that were generated from humanized mice immunized with influenza-specific (P1-5) peptides. (C) HepG2 and JY cells were infected with PR8, X-31, or JAP and used as targets in an ELISpot assay. (D) Additionally, cells were stained for CD8 and CD107a after overnight co-culture and results are given as the mean fluorescence intensity of CD107a gated on CD8+ cells. Results were normalized against unpulsed or uninfected controls.

The neutralization function of anti-M2e antibodies in the serum of peptide-immunized mice was measured by adding serum to influenza infected HepG2 cells. HepG2 cells were infected with a suboptimal dose of PR8 (30HAU/1×10^6^ cells) in the presence of 1∶50 serum. After the initial pulse, serum was added into the culture at a 1∶50 dilution for an overnight incubation. The next day, samples were intracellularly stained for NP as described above.

## Results

### Productive Infection with Influenza Virus for Epitope Discovery

To uncover influenza specific epitopes by immunoproteomics analysis, we chose JY cells and monocyte-derived human DCs as professional APCs, and nonprofessional HepG2 cells, which express the most globally prevalent MHCI molecule, HLA-A*0201 [37] (www.allelefrequency.net). [33]. We first established the infectivity of these cell types *in vitro* by infecting the cells with infectious viral strains A/PR/8/34(H1N1) (PR8), A X-31, Aichi/68(H3N2) (X31), or A2/Japan/305/57(H2N2) (JAP) and assessed nucleoprotein expression [14] (Figure 1). As demonstrated, protein levels were very high, and would prove ideal for immunoproteomic probing and discovery of naturally processed and presented influenza T cell epitopes derived from viral proteins.

**Figure 5:**
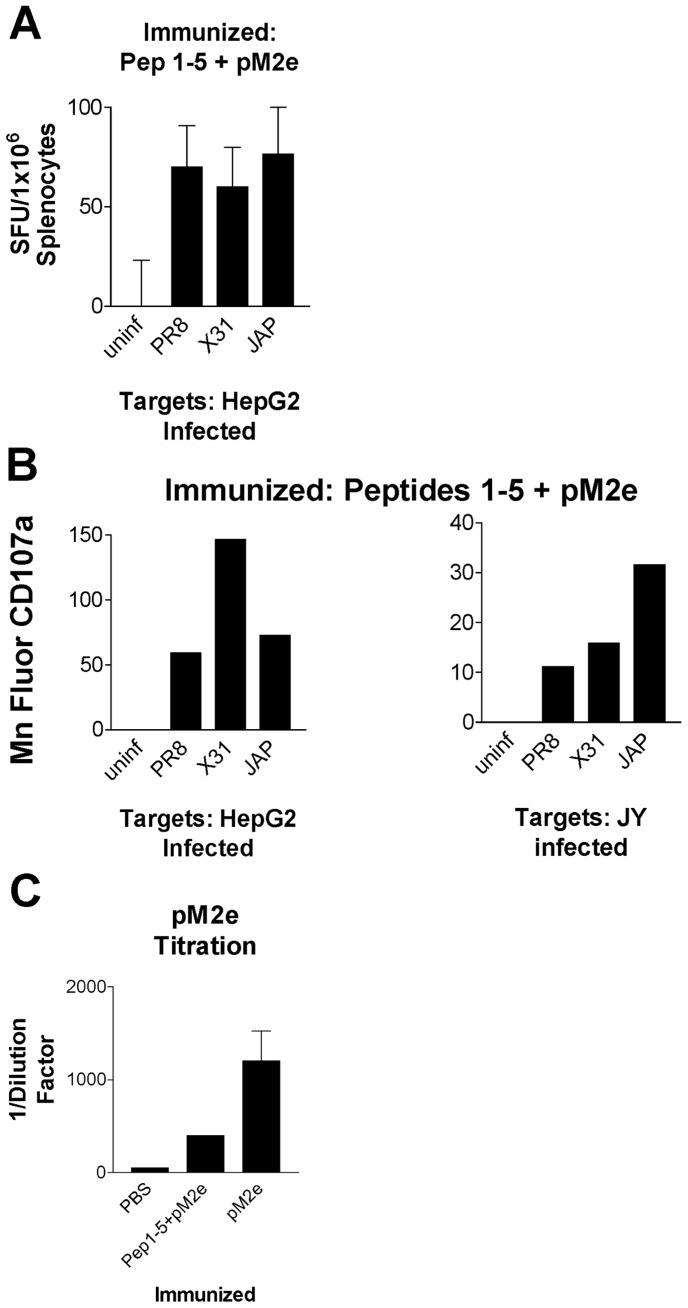
CTLs generated *in vivo* with influenza epitopes in addition to pM2e are specific and cross-reactive. HepG2 and JY cells were infected with PR8, X-31, or JAP and used as targets in an (A) ELISpot assay or (B) FACS analysis using CTLs generated from humanized mice immunized with influenza-specific (P1-5) peptides plus pM2e. (C) Serum titers of mice immunized with PBS, P1-5+pM2e, or pM2e alone were analyzed using ELISA. Plates were coated with pM2e and serum was added and serially diluted. Serum IgG was then detected using anti-mouse IgG secondary reagents. The dilution where the signal was reduced to background was measured and graphed using the reciprocal of the dilution factor (1/DF).

### Identification of Influenza-specific MHC Class I Peptides by Nano-LC/MS/MS Analysis

Employing an immunoproteomics strategy, we identified 6 A2, 10 B44, and 6 B7 motif containing peptides (Table S1). The data including their sequences, virus strains and proteins, HLA-A2, HLA-B44, and HLA-B7 motif anchor residues, and the binding affinity/half time dissociation scores are given in Table S1. Prior to CTL characterization experiments, we confirmed the authenticity of 5 HLA-A2 specific peptides, P1–P5 (Table 1) using their synthetic peptide analogs. As illustrated in Figure 2, most of the fragment ions in the ms/ms spectra of experimentally identified peptides (Figure 2: P1-A–P5-A) matched with spectra of their corresponding synthetic peptides as indicated by the denoted masses (Figure 2: P1-B–P5-B). In the case of P4 peptide, because of the methionine oxidative modification in the synthetic peptide, the corresponding ms/ms spectrum show a mass shift of 16 Da in all the ‘y’ fragment ions (y_5_ and y_6_), precursor ion [(M+H)^+^], and the ions [(M+H)^+^-H_2_O] and [(M+H)^+^-2H_2_O], due to water losses from the precursor ion (Table 1 P4 [B]). In addition, we further verified the ms/ms spectra manually to confirm the identity of all the experimentally observed peptides.

**Figure 6:**
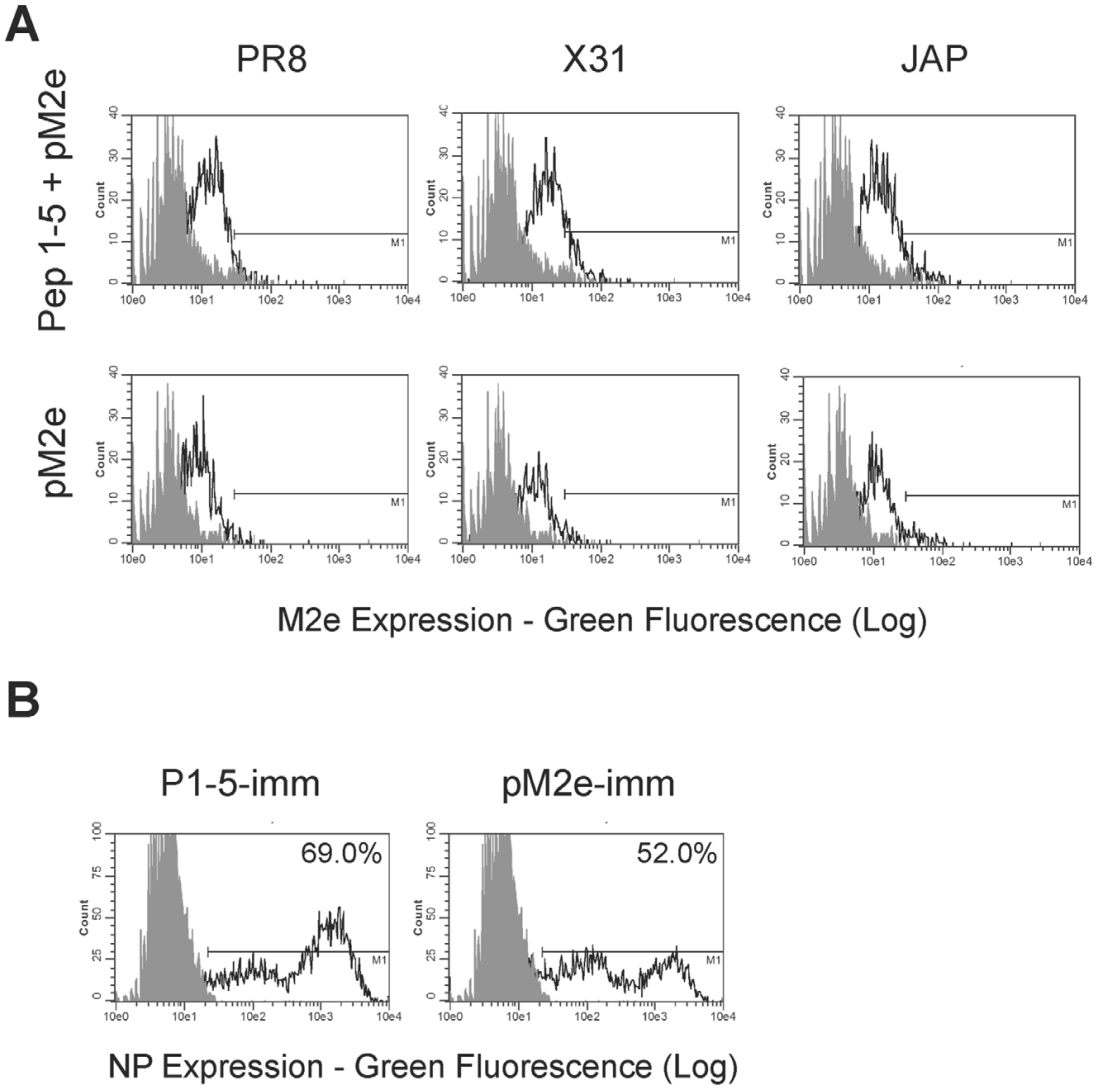
Antibodies against M2e are cross-reactive and neutralizing. (A) HepG2 cells were infected with PR8, X-31, or JAP. After overnight incubation, cells were stained using serum from P1-5+pM2e or pM2e immunized mice at a 1∶50 dilution followed by anti-mouse IgG secondary reagents. (B) HepG2 cells were pulsed with a low dose of PR8 in the presence of serum from P1-5, P1-5+pM2e or pM2e immunized mice at a 1∶50 dilution for 1 hr. Following overnight incubation with the same levels of serum, cells were fixed and intracellularly stained for influenza NP. Percent positive cells are depicted. Gray-filled histograms are uninfected controls.

### CTL Characterization of Influenza Peptides

To verify the presentation of these epitopes by infected cells, CTLs specific for each of the 5 peptides were generated using PBMCs from healthy HLA-A2+ donors and synthetic peptides corresponding to the identified epitopes. In ELISpot assays, CTL functionality was measured by detection of antigen specific IFNγ secretion. As illustrated in Figure 3*A*, PR8-infected JY and HepG2 cells stimulated all five of the influenza epitope-specific T cells. Additionally, cross-reactivity to other strains was demonstrated using HepG2 target cells infected with various influenza A strains (X31, H3N2 and JAP, H2N2), indicating the presentation of these epitopes in various influenza strain-infected cells (Figure 3*B*).

### In vivo Characterization of Influenza Peptides

To further characterize the immune response generated by these epitopes *in vivo*, we immunized HLA-A2+ transgenic mice with a mixture of the aforementioned five epitopes. Immunizations were carried out using these peptides in the presence of Montanide ISA 51 as an adjuvant (Figure 4*A*). We determined the influenza-specific T cell response by measuring murine IFNγ secretion in an ELISpot assay. Using T2 pulsed with individual peptides 1–5, we observed a response to all 5 peptides when mice were immunized with the mixture (Figure 4*B*). In conjunction with above *in vitro* results, *in vivo*-generated CTLs specific for these peptides were stimulated equally well when HepG2 and JY cells infected with various strains of influenza were used as targets (Figure 4*C*) indicating that these epitopes are presented by various influenza strain infected cells.

In addition to IFNγ release, we also measured the phenotypic changes of CD8+ T cells from splenocytes with regards to CD107a, an activation marker present on granulating effector CTLs [35], [36]. As illustrated in Figure 4*D*, splenocytes incubated with infected target cells displayed a higher staining density for CD107a when gated on CD8+ T cells.

### Functional Antibodies to Influenza M2e Peptide

Evaluation of both humoral and T cell immunity simultaneously was accomplished by the injection of multiple T cell epitopes, which drive a strong cellular response, combined with a shared antibody epitope from influenza matrix 2 protein (M2). To this end, we immunized a group of mice with MHCI peptides 1–5 in addition to a peptide from the ectodomain of M2 (pM2e) [17], [18]. To ensure that the T cell response was at least the same as the mice immunized with only P1-5, we repeated our IFNγ ELISpot assay (Figure 5*A*) and CD107a (Figure 5*B*) flow cytometric analysis with the splenocytes from mice immunized with MHCI peptides 1–5+ M2e peptide and observed a comparable T cell response. The concentration of circulating M2e-specific antibody was then measured by a standard ELISA using serum collected from the terminal bleeds of immunized mice. As illustrated in Figure 5*C*, mice immunized with the M2e peptide generated a robust and antigen specific IgG response.

Functionality of the M2e antisera for cross reactivity was then characterized by infecting HepG2 cells with all three strains of influenza virus that we have thus far tested. Using flow cytometry, we labeled the cells with the M2e-specific antisera and demonstrated the binding of the antibody specifically on infected cells indicating the presence of this epitope in various strains of influenza infected cells (Figure 6*A*). Lastly, the neutralizing ability of these antibodies was determined by adding antisera to HepG2 cells infected with PR8 virus. As illustrated in Figure 6*B*, infection levels were lower when the serum containing anti-M2e antibody was used indicating the functional ability of the M2e specific antibodies.

## Discussion

In this study, we attempted to evaluate the immune responses to cross reactive T-cell and antibody epitopes, which together, can work towards the development of a potential universal vaccine. Using an immunoproteomics method, we identified and characterized five naturally presented epitopes that are conserved amongst various strains of influenza virus. Furthermore, we examined the efficacy of these epitopes *in vivo* in combination with a known universal antibody epitope, M2e. We demonstrate a strong T cell response, in addition to neutralization, across several strains. Together, these epitopes could potentially form the backbone of a universal influenza vaccine.

Although current influenza vaccines target humoral immunity, there is evidence that T-cell responses are extremely important for protection against influenza. Importantly, in addition to the role of CTLs in mediating viral clearance [38], [39], CD8+ T cells in humans were shown to have cross-reactive acute [21], [40], [41] and memory responses [42] to different subtypes of influenza A virus. Although our work primarily focused on HLA-A2 supertype-specific T cell epitopes, we have also indicated that other major HLA supertype specific peptides can be identified using the same immunoproteomics approach, which could lead to the generation of a multi-epitope universal vaccine. Goodman *et al* recently demonstrated a multi-epitope DNA prime/pox virus boost recombinant vaccine protected against influenza virus infection in a mouse influenza model [43] indicating this is a valid and effective approach for the generation of a cross-reactive vaccine.

Although a vast number of predicted and immunologically characterized epitopes are reported in the literature (IEDB-AR, http://tools.immuneepitope.org), successful vaccines based on these predicted epitopes have not yet been developed. Generally, motif-based predicted epitopes may activate CTL in the context of *in vitro* assays, however, not all of the activated CTLs will recognize naturally processed antigenic peptides on infected cells due to differences between motif-predicted epitopes and those that are endogenously presented [44]. Furthermore, comparison of the motif prediction method with direct mass spectrometry analysis of endogenously presented epitopes from virus infected cells revealed a high number of predicted epitopes were not processed and presented [22]. Interestingly, we identified a previously reported epitope derived from NS1 protein (P5) [45], which was identified using motif-prediction and *ex vivo* CTL analysis. While this study demonstrates that obtaining an epitope with this approach is possible, it is unlikely to work in a high throughput setting due to the numerous predicted peptide sequences, which vary in their binding affinities for the MHC molecule. Natural presentation of these peptides by infected cells would be difficult to examine without actually analyzing the MHC repertoire.

It is interesting to note that our peptide analysis of influenza infected cells did not yield a well studied motif predicted epitope derived from M1 protein (GILGFVFTL, M1_58–66_) [46]. This is concerning, although informative, and is most likely be due to the fact that this epitope is not well-presented on infected cells. Since epitopes presented by infected cells are critical for generating a clinically relevant protective T cell response, it is likely that this is the reason cellular immunity to seasonal influenza is limited and essentially non-protective [47]. In addition, our epitope specific CTL analysis using PBMCs from healthy donors did not show an enhanced secondary response as one would expect from an influenza-exposed and vaccinated population. Specifically, if memory in the immunized population existed to the peptide epitopes we uncovered, a strong CTL response would have been illustrated. However, the response was moderate at best suggesting no preexisting immunity, i.e. CTL memory, specific for the identified epitopes was present in this population. Our results may also provide reason to believe that epitopes presented by infected cells may be subdominant epitopes [48], [49] and do not activate a strong CTL response as opposed to the dominant epitopes that generate a memory CTL response during infection or vaccination.

The data generated by in silico approaches may overestimate the number of conserved epitopes and may not necessarily identify all immunoreactive naturally presented epitopes. In addition, the motif prediction method may be limited in identifying subdominant epitopes, which are reported to activate T cells in secondary influenza virus specific responses [44]. Furthermore, considering motif predicted epitopes are validated by screening circulating CTLs from virus infected individuals, it is important to note a study by Thomas et al [44] where they demonstrated a CD8+ T cell immunodominance hierarchy that was suggested to be dependent on the concentration of the presented epitope, size of the available CD8+ T cell repertoire, activation after initial priming, and competition and cooperation between different epitope-specific responses. Additionally, Zhong et al. [22] reported a large number of naturally processed and in vivo presented viral CTL epitopes in a mouse model system. In fact, comparison of the motif prediction method with direct mass spectrometry analysis of endogenously presented epitopes isolated from virus infected cells revealed a high number of predicted epitopes were not processed and presented by infected cells [22]. These findings illustrate the complexity of CD8+ T cell based screening of functional epitopes and how this approach may miss hidden subdominant epitopes. Our results, in accordance with these previous studies, further emphasize the need to identify epitopes presented by infected cells and the potential benefit of immunizing individuals with those peptides in a vaccine formulation to gain clinically relevant broad protection.

M2e-specific antibodies have the potential to provide broad protective immunity across influenza A strains. Mice immunized intranasally with combined M2e and T cell determinants exhibited significant production of M2e-specific antibodies as well as decreased morbidity after influenza virus infection [50]. In our study, we have demonstrated strong humoral and cell mediated immunity against influenza virus by immunization with both M2e and five conserved T cell epitopes. We did, however, observe a slight decrease in antibody response when the M2e peptide is immunized in combination with the T cell epitopes. This may be due to the competition of peptide uptake by the APCs. Ultimately, the most promising universal influenza vaccine candidate may come from such a combination of antigens.

In the viral pathogen field, due to the lack of knowledge of vital T cell epitopes, T cell responses to viral infection including influenza have not been routinely assessed in infected patients or in vaccinated individuals. Although several influenza specific T cell epitopes identified by motif prediction and overlapping peptide library screening methodologies exist, a comprehensive analysis of naturally presented T cell epitopes from infected cells, such as our study, has never been undertaken. We believe that these epitopes may serve as part of a universal vaccine candidate complementary to current vaccines and, if successful, could lead to a paradigm shift in how prophylactic and therapeutic antiviral vaccines are formulated.

This report is the first ever on naturally presented conserved influenza specific T cell epitopes identified by direct analysis of infected cells. Additionally, we have demonstrated that conserved epitope-specific CTLs could recognize multiple strain infected target cells and, when combined with a universal antibody epitope, could be useful in a vaccine formulation. Any universal vaccine that produces broad immunity against influenza would be a significant improvement over the current strain-specific vaccines that must be produced anew each year. Moreover, an epitope based vaccine would be produced synthetically, a major improvement over the current method of growing influenza vaccine in fertilized eggs. Therefore, it not only would generate a more effective immune response, but would also prove more economically sensible. The primary objective of this study is an attempt to create such a vaccine product that will complement the universal antibody based vaccines currently in development to combat influenza infection and pandemic.

## Supporting Information

Table S1 **Detailed list of MHC class I presented influenza virus specific peptides.**
(PPTX)
