# Supplementary material for: MHC Class I-Presented T Cell Epitopes Identified by Immunoproteomics Analysis Are Targets for a Cross Reactive Influenza-Specific T Cell Response
Source: PLoS One. 2012 Nov 7;7(11):e48484. doi: 10.1371/journal.pone.0048484 (PMC3492461; doi:10.1371/journal.pone.0048484)
Supplement: Table S1 — Detailed list of MHC class I presented influenza virus specific peptides. (PPTX) [file pone.0048484.s001.pptx]

## Slide 1
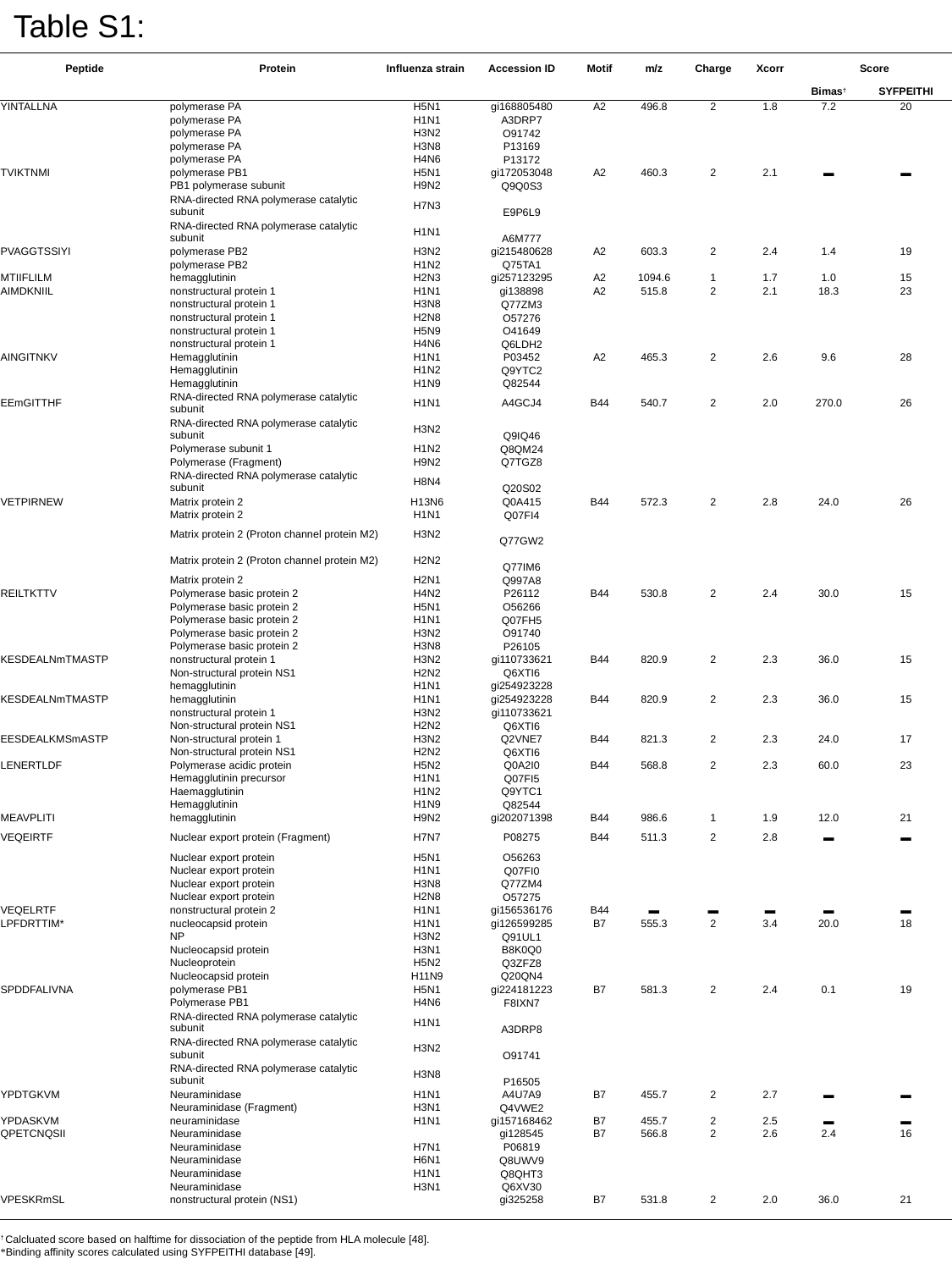

Table S1:
| Peptide | Protein | Influenza strain | Accession ID | Motif | m/z | Charge | Xcorr | Score | |
| --- | --- | --- | --- | --- | --- | --- | --- | --- | --- |
| | | | | | | | | Bimas† | SYFPEITHI |
| YINTALLNA | polymerase PA | H5N1 | gi168805480 | A2 | 496.8 | 2 | 1.8 | 7.2 | 20 |
| | polymerase PA | H1N1 | A3DRP7 | | | | | | |
| | polymerase PA | H3N2 | O91742 | | | | | | |
| | polymerase PA | H3N8 | P13169 | | | | | | |
| | polymerase PA | H4N6 | P13172 | | | | | | |
| TVIKTNMI | polymerase PB1 | H5N1 | gi172053048 | A2 | 460.3 | 2 | 2.1 | ▬ | ▬ |
| | PB1 polymerase subunit | H9N2 | Q9Q0S3 | | | | | | |
| | RNA-directed RNA polymerase catalytic subunit | H7N3 | E9P6L9 | | | | | | |
| | RNA-directed RNA polymerase catalytic subunit | H1N1 | A6M777 | | | | | | |
| PVAGGTSSIYI | polymerase PB2 | H3N2 | gi215480628 | A2 | 603.3 | 2 | 2.4 | 1.4 | 19 |
| | polymerase PB2 | H1N2 | Q75TA1 | | | | | | |
| MTIIFLILM | hemagglutinin | H2N3 | gi257123295 | A2 | 1094.6 | 1 | 1.7 | 1.0 | 15 |
| AIMDKNIIL | nonstructural protein 1 | H1N1 | gi138898 | A2 | 515.8 | 2 | 2.1 | 18.3 | 23 |
| | nonstructural protein 1 | H3N8 | Q77ZM3 | | | | | | |
| | nonstructural protein 1 | H2N8 | O57276 | | | | | | |
| | nonstructural protein 1 | H5N9 | O41649 | | | | | | |
| | nonstructural protein 1 | H4N6 | Q6LDH2 | | | | | | |
| AINGITNKV | Hemagglutinin | H1N1 | P03452 | A2 | 465.3 | 2 | 2.6 | 9.6 | 28 |
| | Hemagglutinin | H1N2 | Q9YTC2 | | | | | | |
| | Hemagglutinin | H1N9 | Q82544 | | | | | | |
| EEmGITTHF | RNA-directed RNA polymerase catalytic subunit | H1N1 | A4GCJ4 | B44 | 540.7 | 2 | 2.0 | 270.0 | 26 |
| | RNA-directed RNA polymerase catalytic subunit | H3N2 | Q9IQ46 | | | | | | |
| | Polymerase subunit 1 | H1N2 | Q8QM24 | | | | | | |
| | Polymerase (Fragment) | H9N2 | Q7TGZ8 | | | | | | |
| | RNA-directed RNA polymerase catalytic subunit | H8N4 | Q20S02 | | | | | | |
| VETPIRNEW | Matrix protein 2 | H13N6 | Q0A415 | B44 | 572.3 | 2 | 2.8 | 24.0 | 26 |
| | Matrix protein 2 | H1N1 | Q07FI4 | | | | | | |
| | Matrix protein 2 (Proton channel protein M2) | H3N2 | Q77GW2 | | | | | | |
| | Matrix protein 2 (Proton channel protein M2) | H2N2 | Q77IM6 | | | | | | |
| | Matrix protein 2 | H2N1 | Q997A8 | | | | | | |
| REILTKTTV | Polymerase basic protein 2 | H4N2 | P26112 | B44 | 530.8 | 2 | 2.4 | 30.0 | 15 |
| | Polymerase basic protein 2 | H5N1 | O56266 | | | | | | |
| | Polymerase basic protein 2 | H1N1 | Q07FH5 | | | | | | |
| | Polymerase basic protein 2 | H3N2 | O91740 | | | | | | |
| | Polymerase basic protein 2 | H3N8 | P26105 | | | | | | |
| KESDEALNmTMASTP | nonstructural protein 1 | H3N2 | gi110733621 | B44 | 820.9 | 2 | 2.3 | 36.0 | 15 |
| | Non-structural protein NS1 | H2N2 | Q6XTI6 | | | | | | |
| | hemagglutinin | H1N1 | gi254923228 | | | | | | |
| KESDEALNmTMASTP | hemagglutinin | H1N1 | gi254923228 | B44 | 820.9 | 2 | 2.3 | 36.0 | 15 |
| | nonstructural protein 1 | H3N2 | gi110733621 | | | | | | |
| | Non-structural protein NS1 | H2N2 | Q6XTI6 | | | | | | |
| EESDEALKMSmASTP | Non-structural protein 1 | H3N2 | Q2VNE7 | B44 | 821.3 | 2 | 2.3 | 24.0 | 17 |
| | Non-structural protein NS1 | H2N2 | Q6XTI6 | | | | | | |
| LENERTLDF | Polymerase acidic protein | H5N2 | Q0A2I0 | B44 | 568.8 | 2 | 2.3 | 60.0 | 23 |
| | Hemagglutinin precursor | H1N1 | Q07FI5 | | | | | | |
| | Haemagglutinin | H1N2 | Q9YTC1 | | | | | | |
| | Hemagglutinin | H1N9 | Q82544 | | | | | | |
| MEAVPLITI | hemagglutinin | H9N2 | gi202071398 | B44 | 986.6 | 1 | 1.9 | 12.0 | 21 |
| VEQEIRTF | Nuclear export protein (Fragment) | H7N7 | P08275 | B44 | 511.3 | 2 | 2.8 | ▬ | ▬ |
| | Nuclear export protein | H5N1 | O56263 | | | | | | |
| | Nuclear export protein | H1N1 | Q07FI0 | | | | | | |
| | Nuclear export protein | H3N8 | Q77ZM4 | | | | | | |
| | Nuclear export protein | H2N8 | O57275 | | | | | | |
| VEQELRTF | nonstructural protein 2 | H1N1 | gi156536176 | B44 | ▬ | ▬ | ▬ | ▬ | ▬ |
| LPFDRTTIM\* | nucleocapsid protein | H1N1 | gi126599285 | B7 | 555.3 | 2 | 3.4 | 20.0 | 18 |
| | NP | H3N2 | Q91UL1 | | | | | | |
| | Nucleocapsid protein | H3N1 | B8K0Q0 | | | | | | |
| | Nucleoprotein | H5N2 | Q3ZFZ8 | | | | | | |
| | Nucleocapsid protein | H11N9 | Q20QN4 | | | | | | |
| SPDDFALIVNA | polymerase PB1 | H5N1 | gi224181223 | B7 | 581.3 | 2 | 2.4 | 0.1 | 19 |
| | Polymerase PB1 | H4N6 | F8IXN7 | | | | | | |
| | RNA-directed RNA polymerase catalytic subunit | H1N1 | A3DRP8 | | | | | | |
| | RNA-directed RNA polymerase catalytic subunit | H3N2 | O91741 | | | | | | |
| | RNA-directed RNA polymerase catalytic subunit | H3N8 | P16505 | | | | | | |
| YPDTGKVM | Neuraminidase | H1N1 | A4U7A9 | B7 | 455.7 | 2 | 2.7 | ▬ | ▬ |
| | Neuraminidase (Fragment) | H3N1 | Q4VWE2 | | | | | | |
| YPDASKVM | neuraminidase | H1N1 | gi157168462 | B7 | 455.7 | 2 | 2.5 | ▬ | ▬ |
| QPETCNQSII | Neuraminidase | | gi128545 | B7 | 566.8 | 2 | 2.6 | 2.4 | 16 |
| | Neuraminidase | H7N1 | P06819 | | | | | | |
| | Neuraminidase | H6N1 | Q8UWV9 | | | | | | |
| | Neuraminidase | H1N1 | Q8QHT3 | | | | | | |
| | Neuraminidase | H3N1 | Q6XV30 | | | | | | |
| VPESKRmSL | nonstructural protein (NS1) | | gi325258 | B7 | 531.8 | 2 | 2.0 | 36.0 | 21 |
| | | | | | | | | | |
| | | | | | | | | | |
| † Calcluated score based on halftime for dissociation of the peptide from HLA molecule [48]. | | | | | | | | | |
| \*Binding affinity scores calculated using SYFPEITHI database [49]. | | | | | | | | | |
